# Health effects of the Federal Bureau of Prisons tobacco ban

**DOI:** 10.1186/1471-2466-12-64

**Published:** 2012-10-15

**Authors:** Stephen A Martin, Bartolome R Celli, Joseph R DiFranza, Stephen J Krinzman, Jennifer G Clarke, Herbert Beam, Sandra Howard, Melissa Foster, Robert J Goldberg

**Affiliations:** 1 Department of Family Medicine and Community Health, University of Massachusetts Medical School, Barre Family Health Center, 151 Worcester Road, Barre, MA, 01005, USA; 2 Harvard Medical School, Brigham and Women’s Hospital, Pulmonary and Critical Care Medicine, 75 Francis Street, Boston, MA, 02115, USA; 3 Department of Family Medicine and Community Health, University of Massachusetts Medical School, 55 Lake Avenue North, Worcester, MA, 01655, USA; 4 Department of Medicine, Division of Pulmonary and Critical Care Medicine, University of Massachusetts Medical School, 55 Lake Avenue North, Worcester, MA, 01655, USA; 5 The Warren Alpert Medical School of Brown University, 111 Brewster Street, CPCP 2nd floor, Pawtucket, RI, 02860, USA; 6 Federal Bureau of Prisons, Federal Medical Center, Devens, P.O. Box 880, Ayer, MA, 01432, USA; 7 Department of Quantitative Health Sciences, University of Massachusetts Medical School, 55 Lake Avenue North, Worcester, MA, 01655, USA

**Keywords:** Pulmonary disease, Chronic Obstructive Pulmonary Disease, Asthma, Pathophysiology, Biomarkers, Pulmonary function tests, Tobacco, Nicotine, Addiction, Health services

## Abstract

**Background:**

Tobacco smoking remains the leading cause of preventable death in America, claiming 450,000 lives annually. Chronic Obstructive Pulmonary Disease, caused by smoking in the vast majority of cases, became the third leading cause of death in the U.S. in 2008. The burden of asthma, often exacerbated by tobacco exposure, has widespread clinical and public health impact. Despite this considerable harm, we know relatively little about the natural history of lung disease and respiratory impairment in adults, especially after smoking cessation.

**Methods/Design:**

Our paper describes the design and rationale for using the 2004 Federal Bureau of Prisons tobacco ban to obtain insights into the natural history of respiratory diseases in adult men and women of different races/ethnicities who are imprisoned in federal medical facilities. We have developed a longitudinal study of new prison arrivals, with data to be collected from each participant over the course of several years, through the use of standardized questionnaires, medical chart reviews, lung function tests, six-minute walk tests, and stored serum for the analysis of present and future biomarkers. Our endpoints include illness exacerbations, medication and health services utilization, lung function, serum biomarkers, and participants’ experience with their health and nicotine addiction.

**Discussion:**

We believe the proposed longitudinal study will make a substantial contribution to the understanding and treatment of respiratory disease and tobacco addiction.

## Background

Tobacco smoking remains the leading cause of preventable death in America and is viewed as “the No. 1 public health concern in the nation and the world” [1-3]. Tobacco’s impact on coronary heart disease and cancer is rivaled only by its role in Chronic Obstructive Pulmonary Disease (COPD), the third leading cause of death in America; smoking cessation and supplemental oxygen remain the only means to reduce mortality for this disease [4]. In 2008, COPD directly accounted for 822,500 hospitalizations among American men and women and was listed as a contributory cause in an additional 3.8 million hospitalizations [5]. In addition to the direct effects of smoking on these illnesses, there is a significant population of patients with asthma and other illnesses who are adversely affected by second-hand smoke [6-8].

Our understanding of the pathophysiology of COPD preceded large-scale tobacco policies, remaining largely informed by the 1960’s study of British industrial workers [9,10]. This prospective study generated the first comprehensive insights into the clinical epidemiology of COPD and established FEV_1_ as the gold standard diagnostic test [11]. Data showing that participants who quit smoking could normalize their rate of lung function decline, but not reverse existing pulmonary damage, was one of this study’s most important findings. This conclusion, however, had significant caveats as findings were based on a relatively small study sample and on self-reported smoking data. In 1976, these and other limitations led the authors to note that “Further studies are needed of the changes of FEV [forced expiratory volume] that occur in obstructed smokers when they stop smoking ….” [12,13].

Despite this need for further research, there have been very few long-term cohort studies of smoking cessation and assessment of its effects on lung health and other disease parameters [14-16]. More recent observational studies have enrolled large numbers of participants and collected comprehensive clinical information [17,18]. Despite the size and breadth of these investigations, early results of these studies highlight our present limitations in understanding lung disease, and question our understanding of its pathogenesis and diagnosis [19]. For example, data published in 2011 from the ECLIPSE study found that in 2,163 COPD subjects, 15% had actual *improvement* in their lung function due to unknown contributory factors [20].

While the costs, constraints, and challenges of long-term observational studies and interventional trials are a serious impediment to understanding the progression of chronic diseases, a natural experiment is currently underway in U.S. prisons. As of 2008, more than half of U.S. states as well as the Federal Bureau of Prisons (BOP) had a total tobacco ban; the BOP ban began in 2004 [21]. Several studies have begun to delineate the impact of these bans. The existing literature, however, has generally focused on characterizing smokers’ addiction and behavior in partial ban settings or over limited periods of time. [22-25]. Our proposed longitudinal study will be able to address a number of important unanswered questions in the field through the use of a study population with and without medical co-morbidities, under a verifiable complete smoking ban over an extended period of follow-up. The present investigation will, for the first time, allow a comprehensive and systematic assessment of the effects of enforced smoking abstinence on respiratory symptoms, pulmonary physiology, biomarkers, and outcomes known to relate to an individual’s long-term survival and health-related quality of life. Though conducted in a prison setting, the subjective, clinical, pulmonary function, and biomarker results would apply to the general population.

We hypothesize our natural history study will find distinct subgroup patterns of improvement, plateau, or decline in terms of health service utilization, lung impairment, and biomarkers. We see this variability as a contrast to the traditional course as interpreted from Fletcher and Peto’s data and more consistent with the ECLIPSE study cited above and recent findings in biomarker patterns [26-29]. If found, such subgroups may allow for the development of targeted therapeutic care.

In addition to information on pulmonary health, the study also allows for considerable impact on public health. With 1% of the U.S. population being incarcerated in a given year, and more than 12% of U.S. smokers released annually from incarceration, exploring a durable means of tobacco abstinence post-release could have a powerful health effect beyond prison [30]. Several studies have shown substantial cardiovascular mortality associated with release from prison; this risk may be due to the high prevalence of tobacco use in the corrections population [31]. Our protocol will allow for a deeper understanding of nicotine addiction in the setting of long-term enforced abstinence and provide for future informed interventions prior to release. Understanding tobacco addiction during and after prison may also help support smoking cessation and prevention efforts in other restricted settings.

## Methods/Design

### Study population

This prospective epidemiologic study will enroll new inmates at Federal Medical Center (FMC) Devens (Devens, MA) and FMC Carswell (Fort Worth, TX) and follow all consenting participants over a five-year period. Devens houses only male inmates and Carswell is the only federal prison medical center for women. FMCs care for the sickest federal inmates; 25% or more of the population at these two facilities are designated as having a high medical need. Other than a disproportionally older population, FMCs have similar demographics to other prisons in their region and are each approximately the same size.

The overall BOP population (N = 216,511) is 38% Black and 59% White, with 35% reporting Hispanic ethnicity. The vast majority of inmates are male (94%) and the average age of this population is 39 years [32]. FMC Devens and FMC Carswell represent populations with considerable race and ethnicity diversity and mirror the general BOP population. While this study represents a poorer, less educated, more minority population than the U.S. as a whole, it does represent the population most impacted by tobacco-related disease [33].

### Participant recruitment

Participants will meet pre-defined inclusion criteria, including:

1) Arriving as a self-surrender (coming from home setting to begin a federal sentence) or as a transfer from a county, state, or other federal prison. In the case of non-federal transfers, note will be made of the tobacco policy of the transferring institution as well as the amount of time the participant was under a tobacco ban;

2) Are anticipated to be in a Bureau prison for at least one year after the initial study start date;

3) Have a history of cigarette smoking of ten or more total pack-years and having smoked within the past two years; and

4) Are able to understand and consent to the study procedures.

Once deemed eligible, each inmate will be asked to provide informed consent and will be enrolled into the study. The study is explained in detail to potential participants, questions answered, they are informed of their rights to quit at any time, and also informed that participation will not influence any prison privileges, probation or parole. If the individual is still interested in participating then the consent form is signed by the inmate. All incentives for participation are prohibited by the BOP. This study is approved by the University of Massachusetts Medical School Institutional Review Board and the Federal Bureau of Prisons Research Review Board.

### Sample size considerations

Sample size was selected to permit analysis of the primary dependent variable, FEV_1_, at an alpha of 0.05 one-tailed and a power level of at least 0.80. A recent systematic review of 47 studies found an expected FEV_1_ decline of 28 mL/year in non-smokers and 33 mL/year in smokers who quit after baseline; a recent observational study of subjects with COPD also found a decline of 33 mL/year over three years [20,34]. Our sample size requirement of approximately 250 participants will be able to detect an average *improvement* in FEV_1_ of 10 mL/year.

### Data collection activities

Participants in this study will complete a detailed baseline questionnaire including participants’ demographic characteristics, lifestyle practices, and smoking history. Standardized and validated measures will also be obtained at intake including: the COPD Assessment Test (CAT), Modified Medical Research Council Dyspnea Scale, the Charlson Self-Report, the Healthy Days Core Module (CDC HRQOL-4), the Autonomy over Smoking Scale (AUTOS), the Hooked on Nicotine Checklist (HONC), the Hughes/Hatsukami Withdrawal Scale, the Questionnaire of Smoking Urges – Brief (QSU-Brief), and the Center for Epidemiologic Studies Depression Scale (CES-D). The four smoking questionnaires will allow comparisons with existing studies. The initial set of questionnaires takes participants less than 1 hour to complete. In the follow-up assessment, designed to take fifteen minutes, only the AUTOS and QSU-Brief questionnaires are administered. Using the standardized survey questionnaires and information obtained from the review of the medical chart, we will longitudinally examine an inmate’s perceived quality of life, reported symptoms, medical diagnoses, medication usage, quantity/cause of prison clinic visits, COPD/asthma exacerbations, quantity/duration of any hospitalizations, and other important related factors.

Study participants’ clinical characteristics will be assessed by conducting a six-minute walk test (6MWT), pre-/post-bronchodilator spirometry, and the diffusion capacity of the lung for carbon monoxide (DLCO) according to American Thoracic Society standards. We will also calculate a BODE index from the body-mass index (B), degree of airflow obstruction (O), dyspnea (D), and exercise capacity (E), measured by the six-minute walk test. This index is a prospectively verified predictor of COPD related mortality [35]. A hand squeeze test will also be administered to measure peripheral muscle strength. Blood and urine samples will be obtained to assess CBC with differential, and levels of C-reactive protein, urine microalbumin, BUN/Cr/GFR, and Hgb A_1_c; these biomarkers have been associated with tobacco consumption and with the development of cardiovascular and respiratory disease. Additional blood will be stored and tested for the presence of nine biomarkers (MMP9, VEGF, IL-1β, IL-6, PARC [CCL18], LTB4, ADAM8, ACE, and CC16) [36]. In a separate process, we will seek informed consent to allow a frozen blood sample to be stored under standard cryogenic conditions for future studies related to tobacco and disease [37]. All personal identifiers will be removed from stored samples.

At defined intervals (initial, 1, 3, and 6 months, 1 year and annually for 5 years total) we will re-administer questionnaires, clinical, respiratory, and serum tests to collect ongoing data on the inmates’ current health status as noted above.

### Time plan

The development of this study began in July of 2009. IRB approval was granted in 2010 and, after further planning, initial enrollment began in March 2011.

### Planned analysis

All collected data will be summarized and presented with the use of simple descriptive statistics using chi square tests to examine differences in various categorical variables (e.g., presence of co-morbid conditions) and t tests to examine differences in selected continuous variables (e.g., physiologic variables) between various comparison groups. Participants will be stratified by how recently they have smoked in our analyses as well as according to the number of cigarettes they smoked and duration of their smoking habit. Longitudinal data analyses will be conducted, including the use of life table methods and person-time approaches, and generalized linear models will be developed using backward stepwise regression methods to identify factors associated with various health related outcomes. We will also utilize interrupted time-series techniques in the analysis of our time-dependent findings.

## Discussion

Pivotal clinical and public health research often takes advantage of unusual circumstances. There has been a growing call for work on the natural history of lung disease and biomarkers and our study offers a high-yield approach [13,14]. Prisons offer a rare opportunity to study a smoke-free community and its impact on human health and illness. Prisons also have populations with significant co-morbidities and health disparities who are important to study [38,39].

This comprehensive assessment, all in the setting of a prison tobacco ban, will provide unique insights into the natural history of respiratory disease in the absence of tobacco. With the storage of biological samples at various lengths of time of tobacco abstinence, we will also be able to examine the effects of smoking cessation on markers of cell injury and repair and potentially identify therapeutic targets for tobacco-related disease. The collaboration with correctional health could also allow for future intervention studies to decrease smoking post-release, with substantial public health benefit [40]. Our study will open important doors for research in tobacco-related pathophysiology, biomarkers, addiction, correctional and public health, health-related quality of life, and health services utilization.

## Abbreviations

COPD: Chronic Obstructive Lung Disease; BOP: Bureau of Prisons; FMC: Federal Medical Center; DLCO: Diffusion Capacity of the Lung for Carbon Monoxide.

## Competing interests

The authors declare that they have no competing interests.

## Authors' contributions

SAM conceived of the study, participated in its design, and helped to draft the manuscript. BRC participated in the study design and helped to draft the manuscript. JRD participated in the study design and helped to draft the manuscript. SJK participated in the study design and helped to draft the manuscript. JGC participated in the study design and helped to draft the manuscript. HB participated in the study design and helped to draft the manuscript. SH participated in the study design and helped to draft the manuscript. MF participated in the study design and helped to draft the manuscript. RJG participated in the study design and helped to draft the manuscript. All authors read and approved the final manuscript.

## Pre-publication history

The pre-publication history for this paper can be accessed here:

http://www.biomedcentral.com/1471-2466/12/64/prepub
